# Perceived Benefits of Yoga among Urban School Students: A Qualitative Analysis

**DOI:** 10.1155/2016/8725654

**Published:** 2016-01-18

**Authors:** Donna Wang, Marshall Hagins

**Affiliations:** ^1^Social Work Department, Long Island University, Brooklyn, Health Science Building 248, 1 University Plaza, Brooklyn, NY 11201, USA; ^2^Department of Physical Therapy, Long Island University, Brooklyn, 1 University Plaza, Brooklyn, NY 11201, USA

## Abstract

This study reports on the findings of a qualitative evaluation of a yoga intervention program for urban middle and high school youth in New York City public and charter schools. Six focus groups were conducted with students who participated in a year-long yoga program to determine their perceptions of mental and physical benefits as well as barriers and challenges. Results show that students perceived the benefits of yoga as increased self-regulation, mindfulness, self-esteem, physical conditioning, academic performance, and stress reduction. Barriers and challenges for a yoga practice include lack of time and space. The extent to which the benefits experienced are interrelated to one another is discussed. Suggestions for future research and school-based programming are also offered.

## 1. Introduction

Yoga is generally accepted as an ancient tradition that incorporates postures, breathing techniques, meditation, and moral and ethical principles [1]. Despite its growing popularity among people of all ages to promote overall health and fitness [2], little is known about the use of yoga among youth [3], especially urban youth. Preliminary quantitative studies have found support for yoga programs improving mood [4], decreasing anger, depression, and fatigue [5], improving stress resilience [6], and reducing problematic physiological and cognitive patterns of response to stress such as rumination, intrusive thoughts, and emotional arousal in fourth and fifth graders in urban public schools [7].

In a systematic review of quantitative yoga studies in schools, Serwacki and Cook-Cottone [8] concluded that although school-based yoga programs appeared to be beneficial, methodological limitations, including lack of randomization, small samples, limited detail regarding the intervention, and statistical ambiguities, prevent the ability to provide definitive conclusions or recommendations. Despite these limitations, the findings thus far support further studies into the benefits of yoga for youth.

In addition to quantitative studies, qualitative studies may add to the understanding of the yoga in schools by describing the process in which the benefits are learned and internalized and thereby informing the practical application as well as the theory underlying yoga programs. To our knowledge there are only two published qualitative studies that examined yoga programs in schools: one in high schools [3] and one in elementary schools [9]. In what Conboy et al. [3] cite as the first published qualitative assessment of yoga benefits for high school settings, it was found that, in a rural public high school, a 12-week yoga program helped students with their athletic performance, bodily awareness, academic performance, sleep, and mental health, such as emotional regulation, stress regulation, and stress reduction. In the qualitative study of elementary school students [9], it was found that an 8-week yoga-based program helped third grade students to feel calm and focused, provided strategies to help control emotions, and supported positive self-esteem.

Given the above, there is currently a lack of published qualitative studies providing information directly from student voices. There is a need to continue to improve understanding of the individual experiences by which yoga programs within school systems may be providing benefits. Therefore, the purpose of the current study was to perform a qualitative assessment using focus groups to examine student responses to the introduction of a yoga program within several middle and high schools in New York City. Unlike previous qualitative assessments of yoga programs in schools, the current study incorporated focus groups from several different schools, was based on a year-long yoga program, and was primarily comprised of minority youth. The overall guiding research questions were as follows: “Do urban youth perceive benefits from learning and practicing yoga? And if so, in what specific ways.”

## 2. Background of Study

During the academic year 2014-2015, the Sonima Foundation (http://www.sonimafoundation.org/) began health and wellness programs in four different NYC schools that primarily serve minority youth. This program was termed a “Health and Wellness” Program, but the program was developed and is congruent with yoga practices; therefore we describe the study as such. The curriculum focuses on mindfulness and yoga-based exercises with the goal of helping students focus on their work and develop the ability to respond appropriately to challenging situations. A typical class begins with a brief seated mental and physical centering exercise followed by teacher led student discussion focusing on the theme/didactic content of the class for that day. Physical postures, breathing exercises, and a final brief relaxation/meditation then followed. The curriculum is manualized and in accordance with the New York State and National Physical Education Standards. Teachers providing the yoga program typically have a minimum of 200-hour training (Registered Yoga Teacher 200) and all attended five days of curriculum training prior to the start of the school year. In addition, during the year (September–June) the teachers received biweekly teacher meetings, professional development workshops (three 2-hour trainings), and a minimum of four classroom observations with feedback.

Implementation of the yoga program varied considerably among the four schools given the unique schedules, physical space, and culture of each school. In general students received one to two class periods of yoga instruction per week across the entire academic year. Selection of students who received yoga was determined by each school's administrators and the selection process varied across schools. School 1 used a cluster randomization of English classes to assign students to the yoga program, while Schools 2, 3, and 4 based assignment on practical concerns such as individual student scheduling or simply nonrandom arbitrary assignment. The demographics of the four schools that implemented the Health and Wellness Program are presented in Table 1. Quantitative studies regarding this program were also implemented and will be reported elsewhere. The quantitative and the current qualitative study were approved by the Long Island University and New York City Department of Education Institutional Review Boards.

## 3. Methods

To examine which physical and mental benefits the students experienced, six semistructured focus groups were conducted at the four sites in May at the conclusion of the school year in which the yoga program had been initiated. An exception was School 1, in which the yoga program had been piloted the previous year. For this school an additional focus group was also held in the fall to capture input from the previous year. School 1 was also unique in that it had multiple ongoing classes of the yoga program. Therefore, two focus groups were conducted in May 2015 at School 1 with each of the classes and thus each of the three focus groups that were conducted at School 1 was of different students.  Table 2 shows information pertaining to how each focus group was conducted.

The purpose of the focus groups was to gather in-depth data from the students about what they thought of the yoga program, perceived physical and mental benefits, and short- and long-term effects of the yoga, as well as challenges to maintaining an ongoing practice (see the Appendix for questionnaire guide). The focus groups lasted approximately 20–40 minutes and were all conducted by one person, who was not affiliated with the yoga study in any other way. Due to school regulations, the focus groups were not audio-recorded but instead had two research assistants take notes throughout. Upon completion of each focus group, each research assistant transcribed and reviewed the notes and sent them to the interviewer for review.

As suggested by Miles and Huberman [10], analysis began with a list of preliminary codes based on the theory and existing literature. These codes are considered provisional until they are “grounded” by being verified by relevant quotes from the focus groups [11]. Transcripts read by the interviewer were initially analyzed using open coding with key phrases noted. After this was completed, based on constant comparative method (CCM) that examines contrasts across respondents, situations, and settings [12], key words and phrases were compared and contrasted and grouped together to form themes in response to the questions that were asked. The contents (or phrases from the focus groups) were then given particular themes.

## 4. Results

The participants were asked to discuss both the mental and physical benefits to practice. There were four main mental health benefits which emerged: self-regulation, mindfulness, reduction of stress, and increased self-esteem. The main physical health benefits which emerged were overall physical conditioning, energy levels, and increased athletic performance.

### 4.1. Self-Regulation

Self-regulation applies to both physical and mental processes. It is the processes in which a system maintains stability of functioning and, at the same time, adapts to new circumstances [13]. Increased self-regulation, or controlling (neutralizing) one's emotions and actions, can be critical to many aspects of life, such as anger control and relationship building. Students freely spoke about how the program helped them to self-regulate and to not take things on a personal level and how to control their reactions and emotions. One student summed it up well when she said “If I go to my big brother and he gets me angry, I go somewhere and calm myself down and then I talk to him respectfully and I tell him why I am angry.” This example shows how students benefit from increasing awareness of anger when it occurs, and how this is linked to the ability to calm down and then improve communication which results in better outcomes. Similarly, another student remarked “yoga taught me the difference between reaction and response” stating that there is a way to feel a reaction and have it not automatically lead to a response. It is interesting to note that a few of the participants stated that they had never previously heard any teaching on how to stop and focus awareness on your mind and control your actions.

Examples given of self-regulation were the following:

“I like the lessons on life … flight or fight and how to control your anger.”

“You do not get upset with people. You can understand the reason behind someone's actions.”

“Yoga helped me a lot. I don't get aggravated as easily. I don't get tense.”

“In real life situations, it taught me to STOP (Stop, Take a breath, Observe the mind, yourself, the problem, breathe, everything going on around you, and Proceed).”

“I now have the knowledge that we can control our anger.”

“I am a really angry person. When you breathe it helps me get through. You cannot act irrationally.”

### 4.2. Mindfulness

According to Kabat-Zinn [13], mindfulness means paying attention in a particular way: on purpose, in the present moment, and nonjudgmentally. Along with self-regulation, students spoke of how yoga in general helped them to change their thinking and become more open-minded, such as understanding other people's perspective, being open to different personalities, and thinking more clearly. This clearer thinking translated into better behavior for one student who stated that it helped her not to act irrationally. Similarly, another student stated that her mother noticed a positive change in her in that the student was approaching situations differently and more mindfully. Another student remarked that yoga helps her to avoid overthinking and therefore overreacting. All these statements reflected a more present-to-present awareness and attention to where the mind goes.

Examples given of mindfulness were the following:

“My focus is better.”

“It helps (me) to understand another's perspective.”

“With yoga you think more clearly … helps you not act irrationally … more tolerant and open to different personalities and it's easier to cross paths with someone and become friends.”

### 4.3. Stress Reduction

Participants articulated how yoga helped them to relax and reduce stress. Often, they discussed how the yoga class was the only place they could do so during the course of the day. They spoke of how the school day consisted of loud people, stressful situations, and constant moving and that yoga helped them to have some mental space for themselves. Often, they spoke of how this state of relaxation carried over for some period of time throughout the day, and even in their lives outside of school, such as with family members and their jobs. One student who worked at a fast food restaurant talked about how “customers are rude” but then I take a breath and it reduced his stress.

Examples given of reduction of stress were the following:

“I want to add to the mental space (continuing from another participant's comment)—I feel like I am the only person in the room. It's so quiet … it's your own personal space.”

“I like it (the yoga)—I am a fast person, being loud—yoga helps me step out of that and relax … put my phone down and relax.”

“I am more aware of the effects of the environment, so now put the shades down at home.”

“Everybody has their bad day but in this environment everything escapes you and it's a new environment for you.”

“The inhale and exhale helped my anxiety. I would do it when I became anxious during the day.”

“The days I don't do yoga, I am hyper. I calm down when I do yoga.”

“Makes me calm and chilled throughout the day, even when I go home.”

“It is helping me outside of school … have to baby sit, cheerleading practice … sometimes stressed out … come back and relax.”

### 4.4. Increased Self-Esteem

Yoga seemed to help increase self-esteem in a number of different ways. First, students who were never exposed to yoga before talked about how they were hesitant to begin, as they were not sure if they could do it. However, many students spoke of being surprised and gaining confidence throughout the year in being able to go deeper and deeper into the poses. This positive experience turned into a life skill for some, as one student remarked “it builds confidence in stressful situations.”

Examples given of increased self-esteem were the following:

“You could be yourself … the one environment and are not graded on your ability to do something … you are just doing something to better yourself.”

“I am capable of doing more now than before.”

“Taught me that I am capable of doing more than I thought I could.”

### 4.5. Improved Physical Conditioning and Energy Levels

Many of the students were involved in sports and other activities that required physical conditioning, such as cheerleading, and many found that yoga complimented their athletic abilities in ways such as helping them stretch better, improved flexibility, and improved balance. In general, increased balance, strength, and flexibility were mentioned repeatedly in all six focus groups among all participants, even those who were not athletes.

Examples given of improved physical conditioning and energy were the following:

“I personally came from a surgery and am recovering. I have gained strength in my left leg … balance poses have actually helped.”

“I use to be more lethargic, and it helped energize me for dancing.”

“I am sleep deprived and the resting made me feel more energized.”

“Yoga has helped me during track practice—before it was really hard but when we started to learn how to deep breathe … now it's easier.”

“It can really help with sports—after a long practice, I come to yoga and it relieves all that stress from the sports when I am sore. I immediately feel better.”

### 4.6. Academic Performance

Another benefit that emerged was that of increased academic performance. Students discussed that the techniques they learned in the yoga programs translated into skills that helped improve their academic performance, such as test taking. For example, students stated “I learned a lot about the brain and nervous system. We have a lot of tests and when I have yoga in the morning it is very calming. It is a way to calm down before the test” and “we learned about deep breathing … so during the test sometimes I take a few breaths if I am struggling.”

### 4.7. Challenges to Practice

Students also discussed challenges and negatives to the program. The main barrier discussed was logistics. Several students mentioned that the urban school setting provides unique challenges to practice, such as not having a dedicated space and not being able to go outside. Another logistic challenge for the students was the noise levels of the school. Even if they had a dedicated space for the yoga class, there was often noise in their immediate proximity. For example, one school held their yoga classes in a room right next to a performance theater. Those practicing yoga would often hear loud music, dancing, and jumping while trying to focus on their class. These may be challenges unique to urban settings and it is possible that schools in other settings may have the resources to address these needs.

Students were asked what they thought would be barriers to continuing an ongoing practice after the school year ended. Interestingly, availability and access were not discussed as an issue. Some of the students already sought out classes in a local yoga studio, and others already found several free classes online that they could use. The barriers that were mentioned were more related to space and time. In terms of a home practice, students mentioned not having space at home to practice, which again may be a by-product of living in tight urban spaces. Students also discussed a lack of available time to continue to practice, which was stated both overtly (i.e., “time … I go to school over the summer … hard to keep up”) and indirectly (i.e., “We like the 5 minute nap which we need” and “you get 45 minutes to relax and mellow down”). Being a high school student can certainly be overwhelming, and thus the time dedicated to help them relax and reduce stress through yoga may be even more necessary than expected.

## 5. Discussion

The results of our study found that high school students perceived the benefits to yoga as increased self-regulation, mindfulness, self-esteem, physical conditioning, academic performance, and stress reduction. One interesting finding is that students were able to translate the teachings and benefits of the yoga practice such as breathing and remaining mindful and in control of their emotions into their lives “off the yoga mat.” For example, increased self-regulation and coping when dealing with family members and school conflicts as well as with academic skills surfaced several times. One student even explicitly stated that “He liked that there were connections to the outside world mentioned during the class” indicating that there were connections realized between the practice and real life situations. Thus it is possible that yoga may increase self-regulation and thus increase life skills. This impact on life off the yoga mat was also echoed in the two previous qualitative studies [3, 9]. A few students mentioned bringing yoga home and teaching their siblings and parents what they had learned. Increasing activities and time together with family in a positive manner may have beneficial outcomes beyond just learning yoga in school.

The findings from the current study are also similar to Case-Smith et al.'s [9] finding that students described benefits as feeling calm and focused, control of stressful situations, and increased self-esteem. These findings are similar despite differences in age of student population, demographics, and specifics of the yoga program. In addition, the results of the current study also found an additional benefit of mindfulness. This additional finding may be due to age or child development (3rd grade versus high school) in that older students may be able to better articulate their perceptions [14]. Other researchers have also suggested that difference in outcomes may occur at different stages and points of development among youth [14]. Thus it is important to bear in mind that what is found for one age group may not necessarily apply to other age groups when considering youth and school-based programming or that similar concepts may be articulated in numerous ways. This reinforces the need for replication of studies for varying demographics.

Our study also supports several of the findings that were found in Case-Smith et al.'s [9] study such as improved athletic performance, use of techniques to help with academic stress, stress reduction, and emotional regulation. However, our study did not find the benefit of bodily awareness that Case-Smith et al. [9] cited, but instead the students in our study articulated concepts related to mindfulness. This difference may be interrelated; for example, mindfulness may impact bodily awareness or vice versa. It could very well be that yoga impacts both, but the differences in the studies presented may also be a result of variation in research methodology, researcher-orientation, or the foci of the yoga programs.

The similarities and differences between the various themes of the qualitative studies are interesting as they provide support for some important concepts such as emotional regulation, stress reduction, and translating teachings to life situations. These specific connections found in three qualitative studies also provide a basis for theory development that could be examined more carefully through both quantitative and continued qualitative inquiry. For example, self-regulation and mindfulness are clearly not mutually exclusive but rather interrelated. One example of this is that it is believed that if you start paying more careful attention from moment to moment, self-regulation naturally happens [13]. However, it is also possible that self-regulation allows for increased mindfulness and attentiveness. It would be beneficial to improve our understanding of the relationship between these two and the pathways in which they affect one another.

In terms of increased academic performance, the participants in this study spoke of how the yoga program developed skills to strengthen their concentration and test-taking abilities. Although it was stated that the students had better test-taking skills, they were less clear regarding whether these skills translated to improved performance on tests. This relationship between benefits of yoga and improved academic performance warrants closer examination in the future as it is critical for school-based programs for at least two reasons. First, the benefits of increased academic performance and athletic ability are important as they may be more concrete (or readily apparent) than some of the more covert mental health benefits. Thus these benefits may be more appealing and immediately attainable and gratifying for some. An attraction to these benefits may be to initially engage an individual in yoga-based programs, and, in return, they may also be exposed to ideas and teachings that are also largely beneficial, such as self-regulation and mindfulness. This strategy of “hiding the peas in the mashed potatoes” is often an effective way of engaging people in activities that they normally may not be interested in. Second, school administration is often interested in the outcomes of increased athletic and academic performance and thus quantitative studies finding these benefits may increase the attractiveness of yoga programs to school administrators.

The challenges to ongoing practice discussed by the students included mostly physical space and time, suggesting that the barriers to practice were logistical. This can be viewed as a positive finding for future yoga programming in that the “complaints” (or resistance) were not based on resistance to the actual practice. As far as time is concerned, this may be an indication that students would benefit from improved time management skills and/or an improved ability to prioritize activities. Yogic philosophy, as well as benefits of stress reduction from yoga, may eventually help encourage these skills and help alleviate time pressures. Again, this is an area of potential future theory development.

There are several limitations to this study. First, the issue of true confidentiality as well as social desirability may have impacted the students' ability to be completely honest and open. Although the yoga teachers and school personnel were not present for any part of the focus groups, the researchers were not affiliated with the actual intervention, and it was stressed that honesty and confidentiality were paramount, there may still have been resistance to speak freely. Likewise, social and peer dynamics may have impacted the responses. Most of these students knew one another, and this may have led to social dynamics that skewed participation and comments in some way.

Another limitation to the study is the manner in which the focus groups were conducted. Although all of the schools were public schools, as aforementioned, each still varied in its governance and culture. Thus, due to the nature of a school setting and regulations, it was not possible to “standardize” the focus groups. For example, the manner in which the participants were invited and selected into the focus groups was completed by the individual school's personnel rather than the researchers. What made most logistical sense and was able to be executed was unique to each school. Another illustration of nonconformity was that some schools had more stringent regulations in regard to informed consent from the students and parents than others, which inadvertently affects who can participate in the study. Yet another example was the inability to get an exact count for focus group #2 as students were constantly coming in and out of the group, which was a typical class period. It is also important to note that high school focus groups are unique in that often we used a class period, and students would view it as such, which translated into several instances of needing to go to the bathroom, getting a drink of water, coming in late, and greeting friends upon arrival. Although this is typical behavior, it also distracted from the flow of the focus groups. Researchers going in to a school often do not know these cultural nuances; thus it is important to consider these issues beforehand and, if possible, discuss with staff the parameters which may affect the process (i.e., not letting anyone enter if they are late). However, this issue is further complicated when staff are not present to help with classroom management for the benefit of confidentiality. As one of the yoga teachers stated, the research intervention has one agenda; however, the school has its own culture and ways of doing things. Thus, researchers should prepare for and attempt to mitigate the inevitable limitations of agency-based evaluation and expect that there will be trade-offs that are necessary to conduct the research.

Despite the above limitations, this current study still adds an important contribution to the qualitative knowledge base of school-based yoga programs as it increases trustworthiness through the increased number of data sources. The ability to include data from four different schools increases the number of perspectives heard, approaching saturation for many of the findings. Another strength of the current study is that the findings were based on an academic year-long program as opposed to shorter interventions. Thus it may be logical that the benefits perceived and skills learned may have crystallized more and have a lasting effect on these individuals. Lastly, the inclusion of a diverse student population provides evidence that these interventions and types of programs may be accessible to various populations.

## 6. Conclusion

This qualitative study of six focus groups across four public schools in New York City found that middle and high school students perceived the benefits to yoga as increased self-regulation, mindfulness, self-esteem, physical conditioning, academic performance, and stress reduction. These findings are congruent with previous published qualitative studies on yoga in schools programs and help to verify and build on past research as well as expand the settings in which these programs are introduced. The results of this study show promising benefits that are both concrete and internalized and which seem to be culturally appropriate for diverse populations as has been suggested to be important [15, 16]. It is hoped that these findings will be helpful to school-based programming and planning as they suggest that these types of programs may be most useful in settings in which self-regulation and stress reduction are problematic.

## Figures and Tables

**Table 1:** Student racial breakdown.

School	Student racial breakdown
School 1	11% Asian, 22% Black, 59% Hispanic, and 8% White
School 2	1% Asian, 62% Black, 32% Hispanic, and 1% White
School 3	0% Asian, 64% Black, 36% Hispanic, and 0% White
School 4	2% Asian, 72% Black, 23% Hispanic, and 2% White

**Table 2:** Focus group (FG) information.

	Location	Date	Grades of participants	Number of participants
FG 1	School 1	September 2014	9–12	6
FG 2	School 2	May 2015	9–12	Approx. 30
FG 3	School 3	May 2015	5–8	7
FG 4	School 1	May 2015	9–12	9
FG 5	School 4	May 2015	9–12	11
FG 6	School 1	May 2015	9–12	11

## Appendix

Focus group interview questions were as follows:

1. What did you think of this year's program?
2. Which aspects of the program did you like best?
3. Which parts did you find most beneficial (different than liking best)?
4. Do you have any interest in keeping up a yoga practice?
5. If not, why not?
6. If you would like to continue, how would you go about doing so? Probe: Find a to community center? On your own at home?
7. If you want to continue, what would be/are some of the barriers?
8. Can you tell me some of the immediate benefits you felt from the yoga?
  - Self-regulation
  - Emotional regulation
  - Academic performance
9. Can you tell me some long-term outcomes or benefits from the program that you are still experiencing?
  - Self-regulation
  - Emotional regulation
  - Academic performance
10. What suggestions do you have to improve this program?
